# Exploratory Analysis of Bedside Variables Associated with Transition Failure in a Selected Cohort of Pediatric Patients Stepped Down from Non-Invasive Ventilation to High-Flow Nasal Cannula After Planned Extubation

**DOI:** 10.3390/jcm15114214

**Published:** 2026-05-29

**Authors:** İbrahim Bingöl, Hacer Uçmak, Kazım Ersin Altınsoy

**Affiliations:** 1 Department of Pediatric Intensive Care, Gaziantep City Hospital, 27470 Gaziantep, Türkiye; ibrahimbingol@gmail.com (İ.B.); prisca.danderfluff@yahoo.com (H.U.); 2 Department of Emergency Medicine, Gaziantep City Hospital, 27470 Gaziantep, Türkiye

**Keywords:** extubation, high-flow nasal cannula, noninvasive ventilation, pediatric intensive care unit, respiratory support transition, ROX index

## Abstract

**Background/Objectives**: High-flow nasal cannula (HFNC) is increasingly used after non-invasive ventilation (NIV) for post-extubation respiratory support in children, but evidence to guide the NIV-to-HFNC step-down decision is limited and the decision itself is heterogeneous across centers. In a hypothesis-generating analysis, we aimed to describe transition failure and explore bedside variables associated with it in a physician-selected cohort of pediatric patients stepped down from NIV to HFNC. **Methods**: This single-center retrospective study included 104 consecutive children (1 month–18 years) extubated, supported with continuous NIV, and stepped down to HFNC. Transition failure was defined as reintubation or re-escalation to NIV within 48 h. Step-down eligibility followed five predefined minimum criteria (Glasgow Coma Scale ≥ 13, FiO_2_ ≤ 50%, SpO_2_ ≥ 90%, hemodynamic stability, and absence of acute hypercapnia); re-escalation and reintubation followed clinical criteria routinely applied in our unit, with the precise timing of each decision left to the attending team. A prespecified exploratory multivariable logistic regression model included the ROX index at transition, the duration of NIV, and the PRISM-III score. Internal validity was assessed by bootstrap optimism-correction, five-fold cross-validation, and leave-one-out cross-validation, with a calibration plot. **Results**: Transition failure occurred in 24 patients (23.1%), with 79.2% within the first 24 h. The ROX index at transition showed the highest univariate discrimination (AUC 0.960, 95% CI 0.916–0.993; cut-off ≤6.0, sensitivity 83.3%, specificity 96.2%). In the exploratory multivariable model, a lower ROX index (adjusted OR 0.10, 95% CI 0.02–0.38; *p* < 0.001) and a longer NIV duration (adjusted OR 1.12 per hour, 95% CI 1.03–1.23; *p* = 0.012) were associated with transition failure; NIV duration likely behaves as a marker of unresolved respiratory pathology rather than a causal risk factor. PICU length of stay, pneumonia, and 28-day mortality were higher in the failure group. **Conclusions**: A transition-moment ROX index ≤ 6.0 and a longer preceding NIV duration were associated with higher risk of step-down failure. These findings are strictly hypothesis-generating, subject to confounding by indication and model optimism, and should not be translated into clinical thresholds before prospective multicenter external validation with pre-specified de-escalation and escalation criteria.

## 1. Introduction

Invasive mechanical ventilation (IMV) is a cornerstone of the management of acute respiratory failure in the pediatric intensive care unit (PICU), yet prolonged IMV is associated with ventilator-associated pneumonia, diaphragmatic dysfunction, and increased mortality. Despite careful readiness assessment, extubation failure continues to occur in 4–22% of pediatric patients and is consistently linked to longer length of stay and higher mortality [1,2]. To reduce the need for reintubation, two forms of non-invasive respiratory support are commonly employed: non-invasive ventilation (NIV), which provides positive pressure and reduces work of breathing, and high-flow nasal cannula (HFNC), which delivers heated and humidified gas at flow rates that generate modest positive end-expiratory pressure and wash out anatomical dead space [3].

Direct comparisons between HFNC and NIV as the primary post-extubation strategy have produced heterogeneous results. Randomized controlled trials in infants with bronchiolitis have shown that HFNC is non-inferior to NIV in mild-to-moderate respiratory distress, while continuous positive airway pressure may be superior in severe cases [4,5]. In children after cardiac surgery, recent meta-analyses reported comparable or slightly lower reintubation rates with HFNC compared with NIV [6]. Overall, neither modality has proven uniformly superior across pediatric indications, and the optimal post-extubation respiratory strategy remains uncertain.

In everyday practice, a commonly adopted approach is a sequential one: patients are first supported with NIV after extubation and subsequently stepped down to HFNC as their respiratory status improves. This de-escalation strategy aims to combine the robust positive-pressure support of NIV in the early post-extubation period with the tolerance and comfort advantages of HFNC later on. A recent international survey of 555 pediatric intensivists confirmed that this approach is widely used but highly heterogeneous, with no standardized criteria for transition [7]. Yet evidence to support the safety and effectiveness of NIV-to-HFNC step-down is scarce, and no pediatric study has specifically identified predictors of HFNC failure at this particular clinical decision point. The Respiratory rate–OXygenation (ROX) index, originally developed in adults with acute hypoxemic respiratory failure and subsequently evaluated in pediatric populations, has emerged as a simple and objective bedside tool for predicting HFNC outcomes. However, its discriminative performance at the specific moment of NIV-to-HFNC transition in children has not been characterized [8,9].

Accordingly, the present study was designed as an exploratory, hypothesis-generating analysis in a selected cohort of pediatric patients who were deemed ready by their attending physicians for step-down from NIV to HFNC after planned extubation. Our aim was not to derive or validate a clinical prediction rule, nor to evaluate the overall effectiveness of the post-extubation strategy itself, but rather to describe the frequency of transition failure within this selected group and to identify bedside variables associated with its occurrence, with a particular focus on the ROX index measured at the moment of transition. We deliberately avoid framing our analysis as a prediction model because of the limited number of failure events, the inevitable confounding by indication inherent to retrospective observational data in which all clinical decisions are physician-driven, and the well-recognized heterogeneity of the step-down decision across pediatric intensive care units. Secondary aims included describing the clinical course of patients with transition failure in terms of PICU and hospital length of stay, ventilator-associated pneumonia, and 28-day mortality. As an additional exploratory analysis, the discriminative performance of individual routinely recorded physiologic parameters—heart rate, respiratory rate, FiO_2_, SpO_2_/FiO_2_, PaCO_2_, and duration of NIV—was also assessed.

## 2. Materials and Methods

### 2.1. Study Design and Setting

This was a single-center, retrospective observational cohort study conducted in the Pediatric Intensive Care Unit (PICU) of Gaziantep City Hospital, a tertiary referral center in southeastern Türkiye. The PICU provides care for critically ill children aged between 1 month and 18 years and is staffed by two attending pediatric intensive care specialists, general pediatricians, and pediatric residents, with 24 h availability of respiratory therapy and invasive and non-invasive ventilatory support. Throughout the 25-month study period, both attending pediatric intensivists shared the overall management of all cases through the unit’s standard on-call rotation; no patient was managed exclusively by a single attending intensivist. Patient records between 1 February 2024 and 28 February 2026 were reviewed. The study was reported in accordance with the Strengthening the Reporting of Observational Studies in Epidemiology (STROBE) statement [9].

### 2.2. Participants

All consecutive patients admitted to the PICU during the 25-month study period who received invasive mechanical ventilation, underwent planned extubation, were subsequently supported with non-invasive ventilation (NIV), and were then stepped down to high-flow nasal cannula (HFNC) were screened for inclusion. The final analytic cohort therefore represents a doubly selected subgroup of all post-extubation patients, namely those in whom NIV was first chosen as post-extubation support and in whom the attending physician subsequently judged the patient ready for step-down to HFNC. Patients who failed extubation very early (reintubation within 1 h) and those with primary upper airway pathology were excluded a priori, which by design removes some of the most severe and earliest post-extubation failures from the analytic cohort. This sequential selection pathway should be considered when interpreting the failure rate, the apparent discrimination of the candidate markers, and the generalizability of the findings.

Inclusion criteria were (i) age between 1 month and 18 years; (ii) receipt of invasive mechanical ventilation during the PICU admission; (iii) planned extubation following a successful spontaneous breathing trial; (iv) initiation of NIV (continuous positive airway pressure or bi-level positive airway pressure) as the post-extubation respiratory support, in accordance with the unit’s standard practice; and (v) subsequent planned step-down from NIV to HFNC.

Exclusion criteria were (i) presence of a tracheostomy; (ii) chronic home invasive or non-invasive respiratory support; (iii) unplanned (accidental or self-) extubation; (iv) reintubation within 1 h of extubation; (v) palliative care plan or a documented do-not-reintubate decision; (vi) primary upper airway pathology including post-extubation stridor, laryngeal edema, or subglottic stenosis; and (vii) severe neuromuscular disease resulting in respiratory pump failure. Patients with missing primary outcome data or key predictor variables were also excluded from the final analysis.

#### Step-Down Decision-Making Process

Because the rationale and practical implementation of the NIV-to-HFNC step-down decision is a central source of heterogeneity in observational studies of post-extubation respiratory support, we describe our local practice in detail. As recently demonstrated in an international survey of 555 pediatric intensivists, post-extubation ventilation strategies vary widely across centers, and no unified protocol has been established for the timing of step-down from NIV to HFNC [7]. NIV was applied as continuous, uninterrupted positive-pressure support after extubation; brief disconnections were permitted only for feeding, oral care, or interface adjustment and were typically shorter than 30 min per episode. Sequential alternation between NIV and HFNC, or between NIV and conventional oxygen therapy, was not used as a weaning strategy in this unit during the study period; once HFNC was initiated, NIV was discontinued. Conventional oxygen therapy was not used as an intermediate step. This continuous approach is consistent with pediatric consensus recommendations on post-extubation noninvasive respiratory support [10] and with adult randomized data on continuous NIV after extubation [11]. In our unit, the following predefined institutional criteria were applied as minimum requirements before any step-down from NIV to HFNC was considered: (i) Glasgow Coma Scale of ≥13; (ii) FiO_2_ requirement of ≤50% on NIV; (iii) peripheral oxygen saturation of ≥90% on the current NIV settings; (iv) stable hemodynamics without escalating vasoactive support over the preceding 6 h; and (v) absence of acute hypercapnia, defined as PaCO_2_ < 50 mmHg with pH ≥ 7.30 on the most recent blood gas. The oxygenation- and consciousness-related thresholds used here are consistent with parameters reported in similar pediatric NIV-to-HFNC studies from comparable regional settings [12,13,14]. During the study period, two patients (1.9%) underwent step-down despite a transition-moment FiO_2_ above this threshold (60% in both cases); these were exceptional decisions made on the basis of overall clinical assessment by the bedside team. Both patients experienced transition failure within 48 h, illustrating the importance of adherence to the predefined oxygenation criterion. These deviations have been retained in the analysis to preserve the integrity of the cohort and are addressed in the Limitations. Decisions to re-escalate from HFNC to NIV, or to proceed to reintubation, were guided by clinical criteria routinely applied in our unit. At present, no published consensus guideline specifically addresses re-escalation or reintubation criteria following HFNC initiation in pediatric patients; the 2023 PALISI international clinical practice guidelines for pediatric ventilator liberation address extubation readiness but do not codify post-HFNC escalation thresholds [15], and published pediatric HFNC studies use heterogeneous operational definitions of failure [13,14]. The criteria used in our unit are therefore framed as a transparent local-practice description rather than as a validated standard. Re-escalation to NIV was considered when, despite optimized HFNC support (flow ≥ 2 L/kg/min and FiO_2_ ≥ 50%), the patient demonstrated persistent SpO_2_ < 90% or sustained tachypnea exceeding the age-adjusted normal range by more than 30%, marked work of breathing with paradoxical respiration, or new-onset hypercapnia (PaCO_2_ ≥ 50 mmHg with pH < 7.30). Reintubation was performed when, despite NIV re-escalation or HFNC, the patient exhibited persistent severe hypoxemia (SpO_2_ < 88% on FiO_2_ 100%), apnea or respiratory arrest, hemodynamic instability requiring vasoactive initiation or escalation, deteriorating consciousness (Glasgow Coma Scale < 12), or inability to manage secretions. The precise timing of these decisions remained at the discretion of the attending team. Throughout the 25-month study period, all step-down, escalation, and reintubation decisions in this unit were made by the same two attending pediatric intensive care specialists (Drs. Bingöl and Uçmak), who also performed the data collection and analysis; this internal homogeneity reduces, although it does not eliminate, the inter-physician variability in the application of the above criteria that typically affects practice in larger multi-attending units. The retention of clinical judgment for the precise timing of step-down, re-escalation, and reintubation within the predefined eligibility windows represents a residual source of confounding by indication that is acknowledged in detail in the Limitations. Single-center retrospective analyses of HFNC failure in Turkish pediatric intensive care units have followed a similar linear NIV-to-HFNC pathway and have been recognized as informative when interpreted within their local-practice context [14].

### 2.3. Clinical Protocol

All patients were managed according to the standard of care of the unit throughout the study period; no study-specific intervention was applied. Extubation readiness was assessed by the attending physician based on resolution of the underlying condition, hemodynamic stability, adequate level of consciousness, acceptable arterial or capillary blood gas values, and a successful spontaneous breathing trial performed with pressure support ventilation. A cuff-leak test was performed in patients considered to be at risk of post-extubation upper airway obstruction.

The post-extubation respiratory strategy followed predefined institutional eligibility criteria for step-down, re-escalation, and reintubation as detailed in the Step-down Decision-Making Process subsection above. The initial HFNC flow rate and FiO_2_ and the subsequent weaning strategy were determined by the attending physician on an individualized basis within these eligibility windows. NIV was delivered as continuous positive-pressure support via appropriately sized oronasal, nasal, or full-face interfaces using conventional mechanical ventilators or dedicated NIV platforms. HFNC was delivered through an integrated heated humidifier system with flow rates initiated at 1.5–2.5 L/kg/minute and FiO_2_ titrated to maintain SpO_2_ between 92% and 97%. While the predefined criteria provided an objective floor for each major clinical decision, the precise timing within the eligible window remained at the discretion of the attending team; this residual clinical discretion is openly addressed in the Limitations.

### 2.4. Data Collection

Patient data were retrospectively extracted from the hospital electronic medical records, bedside nursing charts, and respiratory therapist records into a structured and de-identified electronic case report form. Data collection was performed by two pediatric intensivists using standardized definitions; discrepancies were resolved by consensus.

Data were abstracted at pre-defined clinical moments rather than at fixed calendar-time intervals, reflecting the event-based nature of post-extubation respiratory management. These moments included (1) PICU admission (baseline demographics, diagnosis, severity scores), (2) the invasive mechanical ventilation period (duration, maximum ventilator settings), (3) the moment of extubation (blood gas, cuff-leak test), (4) initiation of NIV (mode, indication, initial settings), (5) the peak NIV support period (maximum IPAP, EPAP, FiO_2_), (6) the moment of transition from NIV to HFNC (defined as the last available measurement on NIV immediately prior to HFNC initiation; this represents the primary evaluation point of the study), (7) HFNC follow-up at clinical decision points (weaning, deterioration, routine reassessment), and (8) HFNC termination (success or failure and reason).

### 2.5. Variables and Outcome Definitions

Baseline variables included age (in months), sex, body weight, height, admission diagnosis, comorbidities, Pediatric Risk of Mortality III (PRISM-III) score, and Pediatric Logistic Organ Dysfunction-2 (PELOD-2) score calculated within the first 24 h of admission [16,17]. Admission diagnoses were categorized into five clinically meaningful groups—bronchiolitis, pneumonia, sepsis, post-operative (including both cardiac and non-cardiac surgery), and other (comprising neurological diseases and miscellaneous conditions)—in a manner consistent with prior pediatric HFNC cohort studies [12].

Invasive mechanical ventilation variables included total duration of mechanical ventilation (in days), maximum positive end-expiratory pressure, maximum FiO_2_, and the last FiO_2_ and arterial/capillary PaCO_2_ recorded immediately prior to extubation. NIV variables included mode, indication (prophylactic, rescue, or post-operative), total duration (in hours), and maximum inspiratory and expiratory positive airway pressures.

To ensure temporal standardization, all transition-moment variables were defined as the last available measurement recorded on NIV immediately before the step-down to HFNC: heart rate (HR), respiratory rate (RR), peripheral oxygen saturation (SpO_2_), FiO_2_, SpO_2_/FiO_2_ ratio, and Glasgow Coma Scale score. The transition-moment PaCO_2_ was defined as the last available arterial or capillary blood gas value obtained while on NIV support. The ROX index was calculated from the transition-moment measurements as (SpO_2_/FiO_2_)/RR, as originally described by Roca et al. [8]. HFNC parameters recorded at initiation included flow rate (in L/kg/minute) and FiO_2_. Biochemical variables (serum albumin, lactate, and C-reactive protein) were obtained closest to the moment of transition.

The primary outcome was transition failure within 48 h of HFNC initiation, defined as reintubation, re-escalation to NIV, or both. Secondary outcomes included reintubation beyond 48 h (late reintubation) during the same PICU admission, total PICU length of stay (LOS), total hospital LOS, ventilator-associated or hospital-acquired pneumonia, grade ≥ 2 nasal trauma, and 28-day mortality. Decisions to reintubate or re-escalate were guided by the clinical criteria described in the Step-down Decision-Making Process subsection above, with the precise timing remaining at the discretion of the attending team.

### 2.6. Statistical Analysis

Continuous variables are presented as mean ± standard deviation or median with interquartile range (IQR) according to distribution, as assessed by the Kolmogorov–Smirnov test. Categorical variables are presented as absolute numbers and percentages. Comparisons between the transition success and transition failure groups were performed using the independent samples *t*-test or Mann–Whitney U test for continuous variables and the chi-square test or Fisher’s exact test for categorical variables.

The discriminative performance of the ROX index at the moment of transition was evaluated as the main analysis using receiver operating characteristic (ROC) curve analysis. As an exploratory analysis, ROC curves were also constructed for each individual physiologic parameter (heart rate, respiratory rate, FiO_2_, SpO_2_/FiO_2_, PaCO_2_, and duration of NIV). For each variable, the area under the curve (AUC) with 95% confidence intervals, optimal cut-off value based on the Youden index, sensitivity, specificity, positive predictive value, and negative predictive value were calculated. AUC values were compared using the DeLong test.

Variables associated with transition failure were explored using a prespecified parsimonious multivariable logistic regression model. The model was specified a priori to include three candidate variables with strong biological and clinical rationale: the ROX index at transition, the duration of NIV, and the PRISM-III score. Variables were not selected based on their univariate performance; this a priori specification was chosen to limit overfitting given the small number of failure events (24), preserving an events-per-variable ratio of approximately 8, and to align with current recommendations for prediction model development in small samples. Heart rate, PaCO_2_, and age—though considered during study planning—were not included in the final model because their univariate discrimination was poor or inconsistent with the prior literature. To mitigate the residual risk of overfitting, the model was fitted using L2-regularized (ridge) logistic regression, and its internal validity was assessed using three complementary procedures: Harrell’s bootstrap optimism correction with 1000 resamples, 5-fold stratified cross-validation, and leave-one-out cross-validation. A uniform shrinkage factor was estimated from the bootstrap procedure as a quantitative summary of expected model optimism. Model calibration was evaluated with the Hosmer–Lemeshow goodness-of-fit test and additionally displayed graphically by a calibration plot of observed versus bootstrap-corrected predicted probabilities of transition failure. We emphasize that, given only 24 events, no internal validation procedure can fully correct for optimism, and the multivariable model is presented as exploratory rather than as a clinically deployable prediction tool. Adjusted odds ratios are reported with 95% confidence intervals. To explore the influence of pediatric age range on the ROX index, a prespecified sensitivity analysis was performed in which the cohort was stratified into children younger than 24 months and children aged 24 months or older, and the area under the ROC curve of the transition-moment ROX index was estimated separately within each stratum. An additional descriptive exploration of transition failure rates and ROX values across the five admission diagnosis categories was performed. Because bootstrap percentile-based confidence intervals for AUC can theoretically exceed the [0, 1] range in small samples near the ceiling of discrimination, any upper confidence limit above 1.000 was truncated to 1.000 in accordance with the theoretical bounds of the AUC metric.

Missing data were handled by complete case analysis. Fifteen of the 132 screened patients (11.4%) were excluded because of missing values in the primary outcome or key predictor variables. Because data on the excluded patients could not be retrieved in detail, a formal comparison of baseline characteristics between included and excluded patients could not be performed; this potential source of selection bias is explicitly acknowledged in the Limitations.

A two-sided *p*-value of less than 0.05 was considered statistically significant. All analyses were performed using IBM SPSS Statistics version 29.0 (IBM Corp., Armonk, NY, USA). The development and reporting of the multivariable model followed the TRIPOD recommendations for transparent reporting of prediction models [18].

### 2.7. Ethics

The study was conducted in accordance with the principles of the Declaration of Helsinki. The protocol was approved by the Clinical Research Ethics Committee of Gaziantep City Hospital (approval no: [483/2026]). Given the retrospective design and the use of de-identified data already recorded as part of routine clinical care, the ethics committee granted a waiver of informed consent. All patient data were anonymized prior to analysis and handled in compliance with institutional data protection policies.

## 3. Results

### 3.1. Study Population and Patient Flow

**Figure 1:**
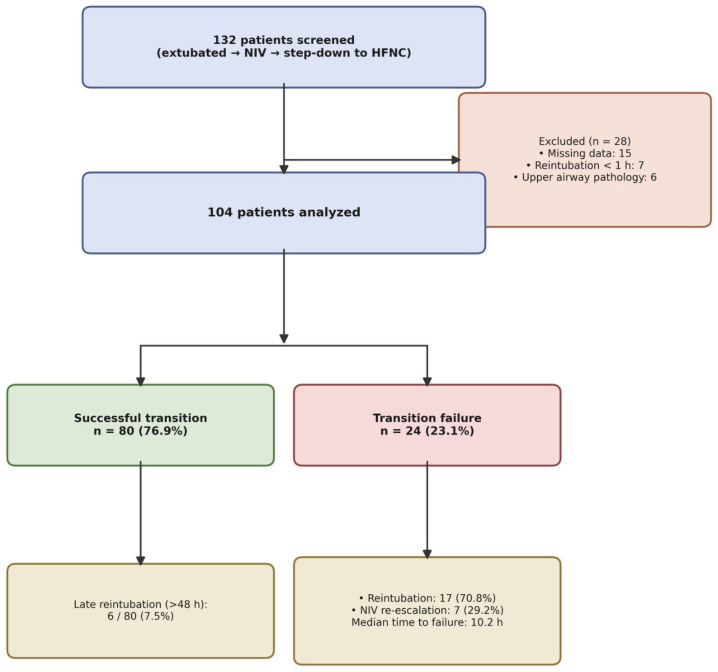
Patient flow diagram of the study cohort. Of 132 pediatric patients who underwent planned extubation, received NIV, and were subsequently stepped down to HFNC during the 25-month study period, 28 patients were excluded (15 due to missing data, 7 due to reintubation within 1 h of extubation, and 6 due to primary upper airway pathology). The final analytic cohort comprised 104 patients, of whom 80 (76.9%) had a successful transition and 24 (23.1%) experienced transition failure (17 required reintubation and 7 were re-escalated to NIV; median time to failure 10.2 h).

During the 25-month study period (1 February 2024–28 February 2026), a total of 132 pediatric patients were identified who underwent planned extubation, received NIV as post-extubation respiratory support, and were subsequently stepped down to HFNC. Of these, 28 patients were excluded: 15 due to missing values in the primary outcome or key predictor variables, seven due to reintubation within 1 h of extubation, and six due to primary upper airway pathology (post-extubation stridor or laryngeal edema). The final analytic cohort comprised 104 patients (Figure 1). It is important to note that the exclusion of the seven patients with very early reintubation and the six patients with upper airway pathology removes some of the earliest and most severe post-extubation failures from the analysis; the results should therefore be interpreted as applying to a selected cohort in whom the attending physician considered NIV-to-HFNC step-down to be a reasonable management pathway.

### 3.2. Baseline Characteristics

The median age of the cohort was 78.0 months (IQR 33.2–138.8), and 59 patients (56.7%) were male. The most frequent admission diagnosis was bronchiolitis (35 patients, 33.7%), followed by pneumonia (25 patients, 24.0%), other diagnoses (20 patients, 19.2%; comprising neurological disease and miscellaneous conditions), sepsis (16 patients, 15.4%), and post-operative conditions (eight patients, 7.7%, including four cardiac and four non-cardiac surgical patients). A comorbidity was present in 39 patients (37.5%), most commonly congenital heart disease. The median PRISM-III score was 12.0 (IQR 9.0–15.0) and the median PELOD-2 score was 5.0 (IQR 3.0–6.0). The median duration of invasive mechanical ventilation prior to extubation was 4.0 days (IQR 3.0–6.0). Following extubation, BiPAP was the predominant NIV mode (99 patients, 95.2%), with CPAP used in the remaining five patients (4.8%). NIV was initiated as rescue therapy in 52 patients (50.0%), prophylactically in 36 patients (34.6%), and in the postoperative setting in 16 patients (15.4%). The median duration of NIV before step-down to HFNC was 25.0 h (IQR 16.0–38.5) (Table 1).

### 3.3. Primary Outcome: Transition Success and Failure

Transition from NIV to HFNC was successful in 80 patients (76.9%) and failed in 24 patients (23.1%) within 48 h of HFNC initiation. Among the 24 patients with transition failure, 17 (70.8%) required reintubation and seven (29.2%) were re-escalated to NIV without reintubation. The median time from HFNC initiation to transition failure was 10.2 h (IQR 6.4–18.6), with 19 of 24 failures (79.2%) occurring within the first 24 h of HFNC.

### 3.4. Comparison of Successful and Failed Transitions

Compared with patients who had a successful transition, those with transition failure were older (median 119.0 vs. 72.5 months, *p* = 0.034), had higher illness severity (PRISM-III 14.5 vs. 11.0, *p* < 0.001; PELOD-2 8.0 vs. 4.0, *p* < 0.001), and had received longer periods of invasive mechanical ventilation (7.5 vs. 4.0 days, *p* < 0.001) with higher maximum PEEP (9.0 vs. 7.0 cmH_2_O, *p* < 0.001) and maximum FiO_2_ (70% vs. 50%, *p* < 0.001).

At the moment of NIV-to-HFNC transition, the failure group had a markedly lower ROX index (5.5 vs. 8.4, *p* < 0.001), a higher respiratory rate (39.5 vs. 30.0/min, *p* < 0.001), higher transition-moment FiO_2_ (50% vs. 30%, *p* < 0.001), and slightly lower Glasgow Coma Scale (14.0 vs. 15.0, *p* < 0.001). Heart rate and transition-moment PaCO_2_ did not differ significantly between groups (*p* = 0.630 and *p* = 0.517, respectively). The total duration of NIV was substantially longer in the failure group (44.0 vs. 23.0 h, *p* < 0.001). Biochemical findings also differed: the failure group had lower serum albumin (2.8 vs. 3.4 g/dL, *p* < 0.001), higher lactate (2.2 vs. 1.1 mmol/L, *p* < 0.001), and higher CRP (86.0 vs. 36.0 mg/L, *p* < 0.001). No statistically significant differences were observed for sex, admission diagnosis category, comorbidity status, NIV mode, or NIV initiation reason (all *p* > 0.05) (Table 2).

### 3.5. ROC Analysis of Predictive Performance

The ROX index at the moment of transition showed the highest discriminative performance for transition failure among all evaluated variables, with an AUC of 0.960 (95% CI 0.916–0.993). At an optimal cut-off value of ≤6.0 (Youden index), sensitivity was 83.3% and specificity was 96.2%.

**Figure 2:**
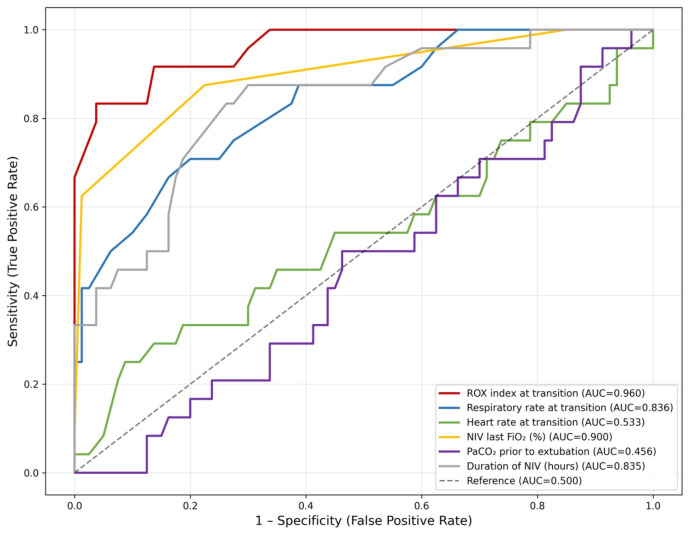
Receiver operating characteristic (ROC) curves of six bedside variables evaluated as predictors of NIV-to-HFNC transition failure in pediatric patients (*n* = 104, 24 failure events). The ROX index at the moment of transition (red) showed the highest discrimination (AUC 0.960), followed by the NIV last FiO_2_ (AUC 0.900), respiratory rate at transition (AUC 0.836), and duration of NIV (AUC 0.835). Heart rate at transition (AUC 0.533) and PaCO_2_ prior to extubation (AUC 0.456) showed no meaningful discrimination.

In the exploratory analysis of individual routinely recorded physiologic parameters, the transition-moment FiO_2_ (AUC 0.900, 95% CI 0.822–0.965; cut-off ≥ 40%), respiratory rate at transition (AUC 0.836, 95% CI 0.741–0.919; cut-off ≥ 36/min), and duration of NIV (AUC 0.835, 95% CI 0.738–0.915; cut-off ≥ 29 h) also showed reasonable discrimination, whereas heart rate (AUC 0.533) and transition-moment PaCO_2_ (AUC 0.456) showed no meaningful discrimination. The ROX index was superior to all other individual parameters by pairwise DeLong testing (all *p* < 0.05) (Table 3, Figure 2).

### 3.6. Multivariable Analysis of Predictors of Transition Failure

In the prespecified parsimonious multivariable logistic regression model containing three candidate variables—the ROX index at transition, the duration of NIV, and the PRISM-III score—a lower ROX index was independently associated with transition failure (adjusted OR 0.097, 95% CI 0.024–0.384; *p* < 0.001), and a longer duration of NIV was also associated with a higher risk (adjusted OR 1.121 per hour, 95% CI 1.025–1.227; *p* = 0.012); the PRISM-III score showed a non-significant trend (adjusted OR 1.531, 95% CI 0.979–2.396; *p* = 0.062) (Table 4). All variance inflation factors were below 2. The NIV-duration estimate is interpreted as a risk marker rather than as an independent causal predictor, given the well-recognized confounding by indication in observational cohorts.

**Figure 3:**
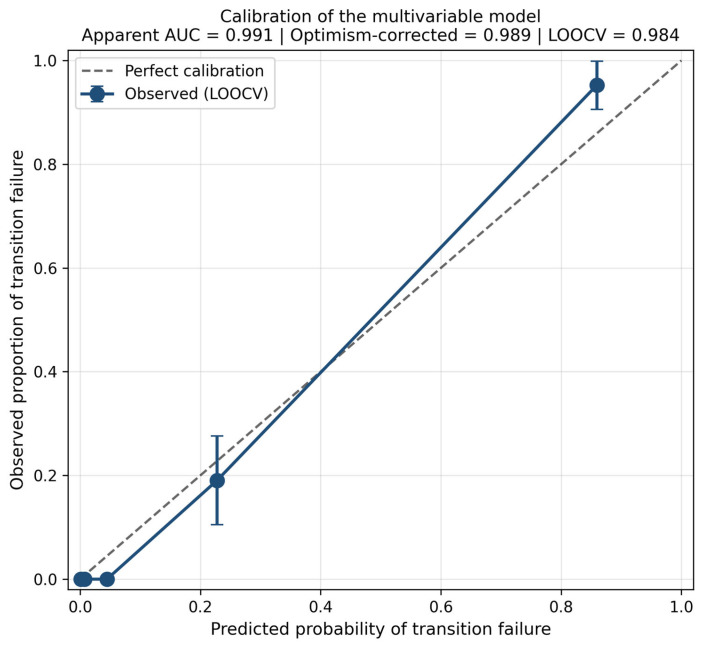
Calibration plot of the prespecified multivariable logistic regression model for NIV-to-HFNC transition failure. The plot displays predicted probabilities of transition failure (x-axis) against observed event proportions (y-axis), estimated by the bootstrap optimism-correction procedure with 1000 resamples. The diagonal reference line represents perfect calibration. Note that with only 24 failure events, the resolution of any calibration estimate is limited.

The apparent AUC of the multivariable model was 0.991 (95% CI 0.974–1.000, upper bound truncated to the theoretical maximum). Internal validation yielded a Harrell’s bootstrap optimism-corrected AUC of 0.989 (mean optimism 0.002), a five-fold cross-validation AUC of 0.988 (SD 0.016), and a leave-one-out cross-validation AUC of 0.984. The bootstrap-based calibration slope was 1.83; the fact that this value exceeds unity reflects the shrinkage already imposed by L2 regularization and indicates that further post-hoc shrinkage is not required. Calibration was adequate by the Hosmer–Lemeshow statistic (χ^2^ = 1.75, *p* = 0.988), and the calibration plot (Figure 3) shows observed proportions closely tracking predicted probabilities across the risk range. The uniformly high AUC estimates partly reflect the small number of events (24), the strong univariate signal of the transition-moment ROX index, and the homogeneous selection of the analytic cohort; residual optimism cannot be excluded, and true external performance is expected to be appreciably lower. The model is therefore reported as an exploratory finding only.

### 3.7. Secondary Outcomes

PICU length of stay (median 17.0 vs. 7.0 days, *p* < 0.001) and total hospital length of stay (23.0 vs. 14.0 days, *p* < 0.001) were significantly longer in the failure group, and ventilator-associated or hospital-acquired pneumonia occurred more frequently (20.8% vs. 5.0%; *p* = 0.029). The 28-day mortality rate was 4.8% in the overall cohort (5/104), with all deaths occurring in the failure group (5/24, 20.8% vs. 0/80; *p* = 0.001). Grade ≥2 nasal trauma was observed in eight patients (7.7%) overall without significant between-group difference (*p* = 0.68). Among patients classified as having successful transitions, six (7.5%) required late reintubation (>48 h) during the same PICU admission (Table 5).

#### Exploratory Subgroup Analyses of the ROX Index

Subgroup ROC analyses of the ROX index across age strata and across major diagnostic categories are summarized in Table 6; given the small subgroup event counts, these analyses are descriptive only and are interpreted in detail in the Discussion.

## 4. Discussion

In this single-center retrospective cohort of 104 children who underwent planned step-down from NIV to HFNC after extubation, transition failure occurred in nearly one-quarter of patients (23.1%), with most failure events clustering within the first 24 h of HFNC initiation. The transition-moment ROX index showed the strongest univariate association with transition failure among all evaluated variables, with an exploratory cut-off of ≤6.0. In a deliberately parsimonious, prespecified multivariable model, both a lower ROX index and a longer duration of preceding NIV were associated with an increased risk of transition failure, although the latter is likely a marker of unresolved respiratory pathology rather than an independent causal factor. Transition failure was accompanied by a higher burden of downstream adverse outcomes, including longer PICU and length of hospital stay, a higher rate of ventilator-associated pneumonia, and a 28-day mortality rate of 20.8% confined entirely to the failure group. We stress at the outset that our cohort represents a physician-selected subgroup of all post-extubation patients; that, although predefined minimum criteria governed eligibility for step-down, re-escalation, and reintubation, the precise timing of these decisions remained at the discretion of the attending team and is therefore subject to residual confounding by indication; and that the very high apparent discrimination of the multivariable model almost certainly contains residual optimism despite multiple internal validation procedures. Our findings should consequently be read as an exploratory, hypothesis-generating description within this specific clinical context rather than as a validated prediction tool or as an evaluation of the effectiveness or safety of the NIV-to-HFNC step-down strategy itself. In light of the substantial international variability in post-extubation ventilation strategies recently documented across 555 pediatric intensivists [7], our description should also be read as a transparent local-practice account rather than as an exemplar of best practice.

The ROX index was originally developed by Roca et al. in adults with acute hypoxemic respiratory failure, in whom a value ≥4.88 at 12 h of HFNC predicted a lower risk of intubation [8]. Pediatric evidence has subsequently emerged but has been heterogeneous with respect to cut-off values. Yıldızdaş et al. proposed a pediatric-adjusted index (p-ROXI) in a mixed population of children with acute respiratory failure and identified different cut-off values at different time points of HFNC therapy [12]. In infants with bronchiolitis, Nascimento et al. reported an optimal ROX cut-off range of approximately 6.5 to 7.2 at 12 h of HFNC, while Kannikeswaran et al. described ROX thresholds between approximately 5 and 8 in a comparable population of bronchiolitis infants requiring positive pressure ventilation [19,20]. In a recent multicenter cohort of 383 infants with bronchiolitis managed in pediatric wards, Le Pallec et al. reported that the ROX index measured at HFNC initiation had only modest overall discrimination for failure and concluded that the index alone was not a reliable single predictor in this setting [21]. Our observed cut-off of ≤6.0 falls within the lower end of the range reported in the pediatric literature and corresponds to a different clinical context: rather than HFNC initiation for primary respiratory support, the ROX was measured at the specific moment of step-down from NIV, when the child had already received a period of positive-pressure support. It is important to acknowledge that our cohort comprises a heterogeneous case mix including bronchiolitis, pneumonia, sepsis, post-operative, and neurological diagnoses. Because the physiology underlying respiratory distress differs across these groups (for example, expected baseline respiratory rate in infant bronchiolitis versus adolescent pneumonia), a single ROX cut-off may not perform identically across all subgroups. This case-mix heterogeneity should be considered a potential limitation on cut-off stability, and disease-specific cut-off values should be evaluated in future larger cohorts. These findings are consistent with the very recent observation by Yuniar et al. that ROX index thresholds of approximately 5.5–5.7 within the first 60–90 min of HFNC identify pediatric patients at high risk of treatment failure [22], supporting the use of lower ROX thresholds in pediatric than in adult cohorts.

A particular caveat is that the ROX index incorporates respiratory rate, a variable whose normal range varies substantially across the pediatric age spectrum. A respiratory rate of 40 breaths per minute is reassuring in a six-month-old recovering from bronchiolitis but profoundly abnormal in a 10-year-old following pneumonia, and any single threshold for ROX is therefore unlikely to perform identically across the wide age range studied here. This is borne out by our prespecified age-stratified sensitivity analysis (Table 6): in children younger than 24 months (*n* = 23 with only two failure events), the transition-moment ROX index yielded an AUC of 0.810 (0.476–1.000), with a wide confidence interval reflecting the small number of events in this subgroup, whereas in children aged 24 months or older (*n* = 81 with 22 failures), the AUC was 0.973 (0.935–0.997), with the Youden-optimal cut-off being ≤7.70 in the younger stratum versus ≤6.60 in the older stratum. Although the wide confidence interval in the younger stratum precludes strong inference, the directional difference in the optimal cut-off is consistent with the concept of age-dependent ROX behavior previously raised by Yıldızdaş et al. in their proposal of a pediatric-adjusted index (p-ROXI) [12]. Disease-specific differences may compound this effect: the physiology of bronchiolitis (small-airway obstruction with elevated respiratory rate), pneumonia (alveolar consolidation with variable respiratory rate), sepsis (systemic inflammation and metabolic acidosis driving compensatory tachypnoea), and the postoperative state (often opioid-related hypoventilation) differ enough that a single ROX cut-off is unlikely to be optimal for all groups. In the descriptive diagnosis-stratified analysis (Table 6, lower panel), Youden-optimal ROX cut-offs ranged from ≤5.20 in postoperative patients to approximately ≤6.60 in sepsis and ≤6.60 in the heterogeneous other category, although the small number of failures within each diagnostic subgroup precludes definitive subgroup inference. Future prospective work in larger pediatric cohorts should explicitly evaluate age-stratified and diagnosis-stratified ROX thresholds and consider whether the index should be reformulated to incorporate age-normalized respiratory rate (for example using age-specific z-scores) before any clinical implementation.

The duration of NIV before step-down emerged as the second variable associated with transition failure in the multivariable model, with each additional hour on NIV corresponding to an approximately 12% increase in the adjusted odds of failure. We deliberately refrain from interpreting this association as an independent causal risk factor. A longer duration of NIV almost certainly acts as a surrogate marker of persistent, unresolved respiratory pathology rather than a causal driver of subsequent HFNC failure: children who require more than a day of positive-pressure support after extubation simply have more severe underlying disease and therefore a higher risk of failure at any subsequent de-escalation step. Furthermore, because the timing of step-down was determined by the attending physician, the observation that longer NIV duration is associated with worse outcomes likely also reflects intuitive deferral of de-escalation in patients perceived as more fragile—a textbook instance of confounding by indication. This source of confounding cannot be removed by statistical adjustment in an observational cohort and represents a fundamental limitation of any prediction analysis derived from physician-driven decisions. The ≥29 h threshold identified in the univariate ROC analysis should therefore be regarded as a candidate severity marker requiring prospective validation and not as a fixed operational rule. Similar observations have been reported by Saelim et al. and by Kamit Can et al. in Turkish PICUs, where prolonged respiratory support and higher severity-of-illness scores were associated with HFNC failure [13,14].

Transition failure was accompanied by a substantial burden of morbidity and mortality. The doubling of PICU length of stay (17 vs. 7 days), the fourfold higher rate of ventilator-associated pneumonia (20.8% vs. 5.0%), and the 28-day mortality of 20.8% in the failure group—compared with no deaths in the success group—highlight the clinical importance of this outcome. These differences should be interpreted descriptively, as the observed burden reflects the overall course of patients who experienced transition failure rather than a causal effect of the failure event itself: patients who fail transition are typically those with more severe underlying illness, and the longer length of stay and higher complication rates represent a combination of the underlying disease trajectory and the direct consequences of reintubation or respiratory re-escalation. The broader literature on pediatric extubation failure reports similar patterns. Kurachek et al. found that reintubation within 48 h was associated with longer hospital length of stay and higher mortality in a multicenter cohort of 2794 children, and Rogerson et al. confirmed these findings in a contemporary analysis of more than 100,000 extubations from the Virtual Pediatric Systems database [1,2]. The finding that late reintubation (>48 h) occurred in 7.5% of “successful” transitions further underscores that the 48 h window does not mark the end of clinical risk.

From a clinical standpoint, our findings should be interpreted with restraint. The simple two-variable bedside combination identified here—a transition-moment ROX index together with the duration of preceding NIV support—provides hypothesis-generating signals that may eventually complement, but must not replace, the bedside judgment of the attending intensivist. We deliberately do not propose ROX ≤ 6.0 or NIV ≥ 29 h as a decision rule, and we caution explicitly against implementing these thresholds in routine care before external prospective validation. Within our own unit, these candidate markers might prompt a more careful reassessment of the de-escalation plan—such as deferring transition pending further respiratory improvement, ensuring closer observation during the high-risk first 24 h of HFNC, or discussing the case with a senior intensivist—but such use should be regarded as informal clinical reasoning rather than as a validated protocol. The high apparent discrimination of the multivariable model in this cohort almost certainly overestimates real-world performance, and transporting these thresholds to another center with a different case-mix, different step-down practices, or a different ratio of failures to successes may yield substantially poorer performance. In line with the TRIPOD recommendations for clinical prediction models [18], our candidate thresholds should not be implemented in routine practice before external prospective validation in independent pediatric cohorts.

Our study has several important limitations. First, it is a retrospective single-center analysis, which limits generalizability to PICUs with different case mixes or institutional protocols. Second, the sequential selection pathway (IMV → planned extubation → NIV → step-down to HFNC) means the cohort represents a doubly selected subgroup rather than a representative sample; the a priori exclusion of seven patients reintubated within 1 h and six patients with primary upper airway pathology removes some of the earliest and most severe post-extubation failures, biases the overall failure rate downward, and inflates the apparent discrimination of the candidate markers. Third, although our unit applied predefined minimum eligibility criteria for step-down, re-escalation, and reintubation (detailed in Methods), the precise timing of each decision within the eligibility window remained at the discretion of the attending team; two minor protocol deviations from the predefined oxygenation criterion occurred (both associated with transition failure). Although decisions were made by only two attending intensivists, attending-level variation in clinical judgment cannot be excluded. This residual clinical discretion creates a substantial risk of confounding by indication and limits any causal interpretation of the multivariable associations. Fourth, although our prespecified parsimonious multivariable model (three candidate variables for 24 events) was subjected to bootstrap optimism correction, five-fold cross-validation, and leave-one-out cross-validation, the limited event count inevitably constrains precision. The exceptionally high optimism-corrected AUC (0.989) should be viewed with explicit skepticism; the bootstrap-based calibration slope of 1.83 indicates that L2 regularization already imposed substantial coefficient shrinkage during model fitting, and true external performance is likely to be appreciably lower [23]. Fifth, the heterogeneity of admission diagnoses precluded robust evaluation of the proposed ROX cut-off across individual disease categories; the descriptive distributions in Table 6 suggest that disease-specific thresholds may be required in larger future studies. Sixth, the ROX index incorporates respiratory rate, which varies with age across the pediatric range; although our age-stratified analysis (Table 6) suggests directionally consistent discrimination, a single ROX threshold may not be optimal across the full age spectrum and an age-normalized reformulation deserves prospective evaluation. Seventh, the ROX index was captured only at the moment of transition and not serially during HFNC therapy; temporal changes may carry additional prognostic information [12]. Eighth, detailed data on the 15 patients excluded for missing values could not be retrieved, so residual selection bias cannot be excluded if missingness was not at random. Ninth, sequential alternation between NIV, HFNC, and conventional oxygen therapy was not used in our unit, so our findings do not apply to centers practicing such alternation. Tenth, outcomes beyond 28 days, including neurodevelopmental sequelae, were not captured. Eleventh, the high mortality concentrated in the failure group (5/24, 20.8%) likely reflects underlying disease severity rather than a direct causal effect of transition failure itself, and the descriptive secondary-outcome comparisons should not be interpreted as evidence that preventing transition failure would necessarily improve survival. Taken together, these limitations imply that our findings should be regarded strictly as hypothesis-generating; prospective multicenter studies with pre-specified de-escalation and escalation criteria, adequate event numbers, and external validation are required before any of the candidate thresholds proposed here can be considered for clinical application.

## 5. Conclusions

In this selected cohort of pediatric patients undergoing planned step-down from NIV to HFNC after extubation, nearly one in four experienced transition failure within 48 h—most within the first 24 h—and failure was accompanied by an appreciable burden of downstream morbidity and mortality. A lower transition-moment ROX index and a longer duration of preceding NIV were the two variables associated with transition failure in a deliberately parsimonious, prespecified multivariable model; the latter is best interpreted as a severity marker rather than as an independent causal predictor. Because this was an exploratory analysis performed in a physician-selected cohort, subject to confounding by indication, to heterogeneity in the step-down decision, and to model performance estimates that are very likely optimistic given only 24 events, our findings are explicitly hypothesis-generating. The candidate thresholds identified here—a transition-moment ROX index of ≤6.0 and a preceding NIV duration of ≥29 h—should not be implemented in clinical practice in their current form. Prospective multicenter studies with pre-specified de-escalation and escalation criteria, adequate event numbers, explicit consideration of age- and diagnosis-specific behavior of the ROX index, and external validation in independent cohorts will be required before these thresholds can be considered for clinical use.

## Figures and Tables

**Table 1:** Baseline characteristics of the study cohort (*n* = 104).

Characteristic	All Patients (*n* = 104)
**Demographics**	
Age (months), median (IQR)	78.0 (33.2–138.8)
Male sex, *n* (%)	59 (56.7)
Weight (kg), median (IQR)	20.0 (11.8–37.5)
**Admission diagnosis, *n* (%)**	
Bronchiolitis	35 (33.7)
Pneumonia	25 (24.0)
Sepsis	16 (15.4)
Post-operative (cardiac + non-cardiac)	8 (7.7)
Other	20 (19.2)
Any comorbidity, *n* (%)	39 (37.5)
**Severity scores at admission**	
PRISM-III, median (IQR)	12.0 (9.0–15.0)
PELOD-2, median (IQR)	5.0 (3.0–6.0)
**Invasive mechanical ventilation**	
Duration of IMV (days), median (IQR)	4.0 (3.0–6.0)
Maximum PEEP during IMV (cmH_2_O)	7.0 (6.0–8.0)
Maximum FiO_2_ during IMV (%)	50.0 (40.0–60.0)
FiO_2_ prior to extubation (%)	30.0 (25.0–35.0)
PaCO_2_ prior to extubation (mmHg)	42.0 (38.0–46.0)
**NIV parameters**	
**NIV mode, *n* (%)**	
BiPAP	99 (95.2)
CPAP	5 (4.8)
**NIV initiation reason, *n* (%)**	
Rescue	52 (50.0)
Prophylactic	36 (34.6)
Post-operative	16 (15.4)
NIV duration (hours), median (IQR)	25.0 (16.0–38.5)
NIV maximum EPAP (cmH_2_O)	8.0 (7.0–9.0)
NIV last FiO_2_ (%)	35.0 (30.0–45.0)
**At the moment of NIV-to-HFNC transition**	
ROX index, median (IQR)	8.0 (6.5–9.6)
Respiratory rate (/min)	32.0 (28.0–38.0)
Heart rate (/min)	122.0 (110.0–138.0)
SpO_2_ (%)	96.0 (94.0–98.0)
Glasgow Coma Scale	15.0 (14.0–15.0)
**HFNC at initiation**	
Flow rate (L/kg/min), median (IQR)	2.0 (1.8–2.3)
Initial FiO_2_ (%)	35.0 (30.0–45.0)
**Biochemistry at transition**	
Serum albumin (g/dL)	3.3 (2.9–3.6)
Serum lactate (mmol/L)	1.4 (1.0–1.9)
C-reactive protein (mg/L)	45.0 (24.0–78.0)

IMV, invasive mechanical ventilation; IQR, interquartile range; NIV, non-invasive ventilation; HFNC, high-flow nasal cannula; PEEP, positive end-expiratory pressure; EPAP, expiratory positive airway pressure; ROX, respiratory rate–oxygenation index; PRISM-III, Pediatric Risk of Mortality III; PELOD-2, Pediatric Logistic Organ Dysfunction-2.

**Table 2:** Univariate comparison of baseline and transition-moment variables between successful and failed transitions.

Variable	Successful (*n* = 80)	Failed (*n* = 24)	*p*-Value
**Demographics**			
Age (months)	72.5 (29.0–127.0)	119.0 (54.5–158.0)	**0.034**
Male sex, *n* (%)	46 (57.5)	13 (54.2)	0.771
Weight (kg)	19.0 (11.0–35.0)	26.5 (15.8–48.5)	0.151
**Admission diagnosis, *n* (%)**			
Bronchiolitis	29 (36.3)	6 (25.0)	0.308
Pneumonia	21 (26.2)	4 (16.7)	0.331
Sepsis	11 (13.8)	5 (20.8)	0.399
Post-operative	5 (6.3)	3 (12.5)	0.366
Other	14 (17.5)	6 (25.0)	0.412
Any comorbidity, *n* (%)	28 (35.0)	11 (45.8)	0.344
**Severity scores at admission**			
PRISM-III	11.0 (8.0–13.0)	14.5 (13.0–18.0)	**<0.001**
PELOD-2	4.0 (3.0–5.0)	8.0 (6.0–9.0)	**<0.001**
**Invasive mechanical ventilation**			
Duration of IMV (days)	4.0 (3.0–5.0)	7.5 (5.0–10.0)	**<0.001**
Maximum PEEP during IMV	7.0 (6.0–8.0)	9.0 (8.0–10.0)	**<0.001**
Maximum FiO_2_ during IMV (%)	50.0 (40.0–55.0)	70.0 (60.0–80.0)	**<0.001**
FiO_2_ prior to extubation (%)	30.0 (25.0–35.0)	35.0 (30.0–40.0)	0.063
PaCO_2_ prior to extubation	42.0 (38.0–46.0)	43.0 (39.0–47.0)	0.517
**NIV parameters**			
NIV duration (hours)	23.0 (15.0–32.0)	44.0 (34.5–56.0)	**<0.001**
NIV maximum EPAP	8.0 (7.0–9.0)	9.0 (8.0–10.0)	**0.012**
NIV last FiO_2_ (%)	30.0 (25.0–35.0)	50.0 (40.0–55.0)	**<0.001**
**At the moment of NIV-to-HFNC transition**			
ROX index	8.4 (7.3–9.9)	5.5 (4.5–6.0)	**<0.001**
Respiratory rate (/min)	30.0 (26.5–35.0)	39.5 (36.0–44.0)	**<0.001**
Heart rate (/min)	120.0 (108.0–138.0)	125.0 (116.0–138.0)	0.630
SpO_2_ (%)	97.0 (94.0–98.0)	95.0 (92.5–97.0)	**0.014**
Glasgow Coma Scale	15.0 (15.0–15.0)	14.0 (13.0–15.0)	**<0.001**
**HFNC at initiation**			
Flow rate (L/kg/min)	2.0 (1.8–2.3)	2.0 (1.9–2.3)	0.632
Initial FiO_2_ (%)	30.0 (25.0–35.0)	50.0 (40.0–55.0)	**<0.001**
**Biochemistry at transition**			
Serum albumin (g/dL)	3.4 (3.1–3.7)	2.8 (2.5–3.1)	**<0.001**
Serum lactate (mmol/L)	1.1 (0.9–1.5)	2.2 (1.8–2.7)	**<0.001**
C-reactive protein (mg/L)	36.0 (20.0–58.0)	86.0 (62.0–118.0)	**<0.001**

Values are shown as median (IQR) or *n* (%). *p*-values are from the Mann–Whitney U test (continuous variables) or Fisher’s exact test (categorical variables). Bold red values indicate *p* < 0.05. PRISM-III, Pediatric Risk of Mortality III; PELOD-2, Pediatric Logistic Organ Dysfunction-2; IMV, invasive mechanical ventilation; PEEP, positive end-expiratory pressure; NIV, non-invasive ventilation; ROX, respiratory rate–oxygenation index; HFNC, high-flow nasal cannula.

**Table 3:** Discriminative performance of transition-moment variables for transition failure (ROC analysis).

Variable	AUC	95% CI	Cut-Off	Sens (%)	Spec (%)	PPV (%)	NPV (%)
ROX index at transition	0.960	0.916–0.993	≤6.0	83.3	96.2	87.0	95.1
NIV last FiO_2_ (%)	0.900	0.822–0.965	≥40	87.5	77.5	53.8	95.4
Respiratory rate at transition	0.836	0.741–0.919	≥36	70.8	80.0	51.5	90.1
Duration of NIV (hours)	0.835	0.738–0.915	≥29	87.5	70.0	46.7	94.9
Heart rate at transition	0.533	0.408–0.656	—	—	—	—	—
PaCO_2_ prior to extubation	0.456	0.326–0.591	—	—	—	—	—

AUC, area under the receiver operating characteristic curve; CI, confidence interval; Sens, sensitivity; Spec, specificity; PPV, positive predictive value; NPV, negative predictive value. NIV, non-invasive ventilation. 95% CIs were estimated by bootstrap resampling (1000 iterations). Optimal cut-offs were selected by the Youden index. Variables with AUC ≤ 0.6 are not reported with cut-off values. The ROX index was superior to all other individual parameters by the DeLong test (all *p* < 0.05).

**Table 4:** Prespecified multivariable logistic regression model for transition failure (*n* = 104; 24 events).

Variable	Adjusted OR	95% CI	*p*-Value
ROX index at transition (per 1-unit increase)	0.097	0.024–0.384	**<0.001**
Duration of NIV (per 1 h increase)	1.121	1.025–1.227	**0.012**
PRISM-III score (per 1-point increase)	1.531	0.979–2.396	0.062

The model was fitted using L2-regularized (ridge) logistic regression with predictors prespecified a priori. OR, odds ratio; CI, confidence interval. PRISM-III, Pediatric Risk of Mortality III; NIV, non-invasive ventilation; ROX, respiratory rate–oxygenation index. Bold red values indicate *p* < 0.05.

**Table 5:** Secondary clinical outcomes by transition status.

Outcome	Successful (*n* = 80)	Failed (*n* = 24)	*p*-Value
PICU length of stay (days), median (IQR)	7.0 (5.0–10.0)	17.0 (12.0–24.0)	**<0.001**
Hospital length of stay (days), median (IQR)	14.0 (10.0–19.0)	23.0 (17.0–32.0)	**<0.001**
VAP/HAP, *n* (%)	4 (5.0)	5 (20.8)	**0.029**
28-day mortality, *n* (%)	0 (0.0)	5 (20.8)	**0.001**
Grade ≥ 2 nasal trauma, *n* (%)	6 (7.5)	2 (8.3)	0.680
Late reintubation (>48 h), *n* (%)	6 (7.5)	0 (0.0)	—

IQR, interquartile range; PICU, pediatric intensive care unit; VAP/HAP, ventilator-associated/hospital-acquired pneumonia. *p*-values are from the Mann–Whitney U test (continuous variables) or Fisher’s exact test (categorical variables). Bold red values indicate *p* < 0.05. The *p*-value for late reintubation is not reported because of a zero cell in the failure group.

**Table 6:** Exploratory subgroup performance of the transition-moment ROX index by age stratum and admission diagnosis.

Subgroup	*n*	Failures, *n* (%)	ROX AUC (95% CI)	Cut-Off (Youden)
Age stratum				
<24 months	23	2 (8.7)	0.810 (0.476–1.000)	≤7.70
≥24 months	81	22 (27.2)	0.973 (0.935–0.997)	≤6.60
Admission diagnosis				
Bronchiolitis	35	6 (17.1)	0.949 (0.839–1.000)	≤6.00
Pneumonia	25	4 (16.0)	0.945 (0.815–1.000)	≤5.80
Sepsis	16	5 (31.3)	1.000 (1.000–1.000)	≤6.60
Post-operative	8	3 (37.5)	1.000 (1.000–1.000)	≤5.20
Other	20	6 (30.0)	0.977 (0.893–1.000)	≤6.60

AUC, area under the receiver operating characteristic curve; CI, confidence interval. Subgroup analyses are exploratory and limited by small numbers of failure events within each stratum; results should be interpreted as descriptive only. The wide confidence interval in the <24-month stratum (only 2 failure events) and the AUC reaching the theoretical maximum of 1.000 in the sepsis and post-operative subgroups (≤5 events each) underscore that these estimates are unstable and not suitable as fixed clinical thresholds.

## Data Availability

Data available upon request from the authors.

## Abbreviations

The following abbreviations are used in this manuscript:

AUC	area under the receiver operating characteristic curve
BiPAP	bilevel positive airway pressure
CI	confidence interval
CPAP	continuous positive airway pressure
CRP	C-reactive protein
EPAP	expiratory positive airway pressure
FiO_2_	fraction of inspired oxygen
GCS	Glasgow Coma Scale
HFNC	high-flow nasal cannula
HAP	hospital-acquired pneumonia
HR	heart rate
IMV	invasive mechanical ventilation
IPAP	inspiratory positive airway pressure
IQR	interquartile range
LOS	length of stay
NIV	non-invasive ventilation
NPV	negative predictive value
OR	odds ratio
PaCO_2_	partial pressure of carbon dioxide
PEEP	positive end-expiratory pressure
PELOD-2	Pediatric Logistic Organ Dysfunction-2
PICU	pediatric intensive care unit
PPV	positive predictive value
PRISM-III	Pediatric Risk of Mortality III
ROC	receiver operating characteristic
ROX	Respiratory rate–OXygenation index
RR	respiratory rate
SD	standard deviation
SpO_2_	peripheral oxygen saturation
STROBE	Strengthening the Reporting of Observational Studies in Epidemiology
VAP	ventilator-associated pneumonia

## References

[1] Kurachek S.C., Newth C.J., Quasney M.W., Rice T., Sachdeva R.C., Patel N.R., Takano J., Easterling L., Scanlon M., Musa N. (2003). Extubation failure in pediatric intensive care: A multiple-center study of risk factors and outcomes. Crit. Care Med..

[2] Kim F.Y., Soto-Campos G., Palumbo J., Newth C.J., Rice T.B. (2025). Extubation Failure in the PICU: A Virtual Pediatric Systems Database Study, 2017–2021. Pediatr. Crit. Care Med..

[3] Venanzi A., Di Filippo P., Santagata C., Di Pillo S., Chiarelli F., Attanasi M. (2022). Heated Humidified High-Flow Nasal Cannula in Children: State of the Art. Biomedicines.

[4] Maya M., Rameshkumar R., Selvan T., Delhikumar C.G. (2024). High-Flow Nasal Cannula Versus Nasal Prong Bubble Continuous Positive Airway Pressure in Children With Moderate to Severe Acute Bronchiolitis: A Randomized Controlled Trial. Pediatr. Crit. Care Med..

[5] Santos A.C.E.Z., Caiado C.M., Lopes A.G.D., de França G.C., Eisen A.K.A., Oliveira D.B.L., de Araujo O.R., de Carvalho W.B. (2024). Comparison between high-flow nasal cannula (HFNC) therapy and noninvasive ventilation (NIV) in children with acute respiratory failure by bronchiolitis: A randomized controlled trial. BMC Pediatr..

[6] Shahzad M., Alharthy M., Alanazi W., Alanazi A., Alanazi F., Alzahrani H., Alharbi H., Alsubaie S., Almoutaz A., Aldughaythir M.S. (2025). Comparative Effectiveness of High-Flow Nasal Cannula Versus Non-Invasive Ventilation for Post-Extubation Respiratory Support After Pediatric Cardiac Surgery: A Systematic Review and Meta-Analysis. Cureus.

[7] Loberger J.M., Campbell C.M., Colleti J., Borasino S., Abu-Sultaneh S., Khemani R.G. (2022). Pediatric Ventilation Liberation: A Survey of International Practice Among 555 Pediatric Intensivists. Crit. Care Explor..

[8] Roca O., Caralt B., Messika J., Samper M., Sztrymf B., Hernández G., García-De-Acilu M., Frat J.-P., Masclans J.R., Ricard J.-D. (2019). An Index Combining Respiratory Rate and Oxygenation to Predict Outcome of Nasal High-Flow Therapy. Am. J. Respir. Crit. Care Med..

[9] von Elm E., Altman D.G., Egger M., Pocock S.J., Gøtzsche P.C., Vandenbroucke J.P., STROBE Initiative (2007). The Strengthening the Reporting of Observational Studies in Epidemiology (STROBE) statement: Guidelines for reporting observational studies. Lancet.

[10] Carroll C.L., Napolitano N., Pons-Òdena M., Iyer N.P., Korang S.K., Essouri S. (2023). Second Pediatric Acute Lung Injury Consensus Conference (PALICC-2) of the Pediatric Acute Lung Injury and Sepsis Investigators (PALISI) Network. Noninvasive Respiratory Support for Pediatric Acute Respiratory Distress Syndrome: From the Second Pediatric Acute Lung Injury Consensus Conference. Pediatr. Crit. Care Med..

[11] Ferrer M., Valencia M., Nicolas J.M., Bernadich O., Badia J.R., Torres A. (2006). Early noninvasive ventilation averts extubation failure in patients at risk: A randomized trial. Am. J. Respir. Crit. Care Med..

[12] Yildizdas D., Yontem A., Iplik G., Horoz O.O., Ekinci F. (2021). Predicting nasal high-flow therapy failure by pediatric respiratory rate-oxygenation index and pediatric respiratory rate-oxygenation index variation in children. Eur. J. Pediatr..

[13] Saelim K., Thirapaleka B., Ruangnapa K., Prasertsan P., Anuntaseree W. (2022). Predictors of high-flow nasal cannula failure in pediatric patients with acute respiratory distress. Clin. Exp. Pediatr..

[14] Kamit Can F., Anil A.B., Anil M., Zengin N., Durak F., Alparslan C., Göl Serin H. (2018). Predictive factors for the outcome of high flow nasal cannula therapy in a pediatric intensive care unit: Is the SpO_2_/FiO_2_ ratio useful?. J. Crit. Care.

[15] Abu-Sultaneh S., Iyer N.P., Fernández A., Gaies M., González-Dambrauskas S., Hotz J.C., Kneyber M.C.J., López-Fernández Y.M., Rotta A.T., Werho D.K. (2023). Executive Summary: International Clinical Practice Guidelines for Pediatric Ventilator Liberation, A Pediatric Acute Lung Injury and Sepsis Investigators (PALISI) Network Document. Am. J. Respir. Crit. Care Med..

[16] Pollack M.M., Patel K.M., Ruttimann U.E. (1996). PRISM III: An updated Pediatric Risk of Mortality score. Crit. Care Med..

[17] Leteurtre S., Duhamel A., Salleron J., Grandbastien B., Lacroix J., Leclerc F., Groupe Francophone de Réanimation et d’Urgences Pédiatriques (GFRUP) (2013). PELOD-2: An update of the PEdiatric logistic Organ Dysfunction score. Crit. Care Med..

[18] Collins G.S., Reitsma J.B., Altman D.G., Moons K.G.M. (2015). Transparent Reporting of a multivariable prediction model for Individual Prognosis or Diagnosis (TRIPOD): The TRIPOD Statement. BMJ.

[19] Nascimento M.S., Zólio B.A., Vale L.A.P.A., Silva P.A.d.L., Souza T.S., Gonçalves L.H.R., Fascina L.P., Prado C.D. (2024). ROX index as a predictor of failure of high-flow nasal cannula in infants with bronchiolitis. Sci. Rep..

[20] Kannikeswaran N., Whittaker P., Sethuraman U. (2022). Association between respiratory rate oxygenation index and need for positive pressure ventilation in children on high flow nasal cannula for bronchiolitis. Eur. J. Pediatr..

[21] Le Pallec C., Cerasuolo D., Cauvin J.C., Agossah C., Milesi C., Savy N., Brossier D.W. (2025). Infants with bronchiolitis with high-flow nasal cannula in the paediatric ward. Is there a role for the ROXi (respiratory rate–oxygenation index) in predicting failure of high-flow nasal cannula?. Eur. J. Pediatr..

[22] Yuniar I., Pudjiadi A.H., Dewi R., Prawira Y., Puspaningtyas N.W., Tartila T., Fulki S. (2024). Respiratory Rate Oxygenation (ROX) index as predictor of high flow nasal cannula in pediatric patients in pediatric intensive care unit. BMC Pulm. Med..

[23] van Smeden M., Moons K.G.M., de Groot J.A.H., Collins G.S., Altman D.G., Eijkemans M.J.C., Reitsma J.B. (2019). Sample size for binary logistic prediction models: Beyond events per variable criteria. Stat. Methods Med. Res..

